# Stunting of children under two from repeated pregnancy among young mothers

**DOI:** 10.1038/s41598-020-71106-7

**Published:** 2020-08-31

**Authors:** Joemer Calderon Maravilla, Kim Betts, Linda Adair, Rosa Alati

**Affiliations:** 1 Institute for Social Science Research, The University of Queensland, Brisbane, QLD Australia; 2 Life Course Centre, Australian Research Council Centre of Excellence for Children and Families Over the Life Course, Brisbane, Australia; 3 School of Public Health, Curtin University, Perth, Australia; 4 Carolina Population Center, University of North Carolina at Chapel Hill, Chapel Hill, NC USA; 5 Institute of Nursing, Far Eastern University, Manila, Philippines

**Keywords:** Risk factors, Epidemiology, Outcomes research, Paediatric research

## Abstract

Repeated pregnancy leaves young mothers nutritionally deprived which may in turn lead to poor infant growth. We measure the occurrence and persistence of stunting among offspring of young mothers who experienced repeated pregnancies using data from the Cebu Longitudinal Health and Nutrition Survey. We selected mothers aged 14–24 years (n = 1,033) with singleton birth. We determined the length-for-age z scores (LAZ) at 12 and 24 months of the index child using the World Health Organisation 2007 growth standard. We fitted LAZ, stunting occurrence (i.e. LAZ < − 2) and persistence from 12 to 24 months into regression models and tested for the mediating effect of low birthweight and feeding practices. In these models, repeated pregnancy was analysed in an ordinal approach using number of past pregnancies of young mothers at birth of the index child. Compared to infants born to young mothers aged 14–24 years who had no previous pregnancies, those born to young mothers with repeated pregnancies have at least 0.15 (95% CI − 0.23, − 0.08) LAZ lower and are at higher chance of stunting by at least 40% (95% CI 1.19, 1.67) at 12 and 24 months. Similar cohorts of infants showed an elevated risk of persistent stunting from 12 through 24 months with a relative risk ratio of 1.51 (95% CI 1.21, 1.88). Optimal feeding practices substantially mediated stunting outcomes by further reducing the effects of repeated pregnancy to stunting occurrence and persistence by 19.95% and 18.09% respectively. Mediation tests also showed low birthweight in the causal pathway between repeated pregnancy and stunting. Repeated pregnancy in young mothers is a predictor of stunting among children under 2 years. Secondary pregnancy prevention measures and addressing suboptimal feeding practices are beneficial to mitigate the negative impact of repeated adolescent pregnancy on children.

## Introduction

Globally, stunting affects more than 100 million children under five^1^, and is associated with poor cognition, reduced school performance, immunodeficiency, and child mortality^2^. In addition to adverse health outcomes, stunted children tend to have poorer economic productivity and lower wages in adulthood^3^. These negative impacts make stunting, especially in ‘the first 1,000 days’^4^, a profound indicator of poor health, social inequality, and disadvantage.

The pathogenesis of stunting originates in the first 1,000 days, extending from early foetal development to 24 months after birth. Inadequate maternal nutrition and poor antenatal care can directly and indirectly result in an unhealthy intrauterine environment and poor foetal growth^5^. Immediately following birth, suboptimal infant feeding practices slow offspring’s growth rate^5^. For example, sub-optimal (i.e. late, inadequate and inappropriate) complementary feeding negatively affects infant nutrition due to the rapid increase in nutritional needs after 6 months of age. Diarrheal infections and hygiene practices related to poor socio-economic status (SES) can also lead to stunting due to nutrient malabsorption and high intestinal permeability^6^.

Early pregnancies play an important role in stunting, due the competing demands for young mothers’ pubertal development and the growth of the foetus^7, 8^. This leads to greater nutrition partitioning, which compromises the development of both mothers and foetus^7, 8^. A repeated pregnancy in adolescence aggravates this mechanism through further depletion of nutritional stores. This may result in preterm births, maternal complications, and low birthweight, which are in turn strong risk factors for offspring stunting^1, 9^.

Although current research indicates the impact of repeated pregnancy among young mothers on child stunting, there is a lack of rigorous evidence in support of this relationship. An analysis of prospective cohorts in developing countries showed lower length-for-age *z* scores (LAZ) at 24 months among offspring of 14- to 19-year-old mothers compared to older age groups^10^. In another study, an unadjusted correlation was observed in this study between LAZ and a parity of 2 or more^10^. On the other hand, a cross-sectional study revealed null associations between infant stunting and parity despite diminishing LAZ in an increasing parity score based on crude data^11^. These inconsistent findings call for the need to explore the impact of parity on stunting trajectories between 12 and 24 months. Trajectories indicating either persistence or recovery, especially during the peak age for stunting, may provide important information about long-term offspring outcomes.

We sought to explore the growth trajectories of the subsequent offspring of young mothers in the Philippines. As a developing country, the Philippines is an ideal site to explore this research question for two main reasons. Firstly, the Philippines has a high rate of fertility in young women compared to other low- and middle-income countries^12^. Secondly, one third of pregnant Filipino adolescents are undernourished^13^, which predisposes a high number of their children to poor nutrition.

In this study, we aim to measure the magnitude of the association between repeated pregnancy in young mothers and offspring stunting at 12 and 24 months, and its persistence from 12 up to 24 months. Our study also explores the potential mediating effect of low birthweight, as proxy to evaluate foetal growth and nutrition, and feeding practices at significant timepoints to further investigate the modifiability of stunting risks introduced by having repeated pregnancies (see S1). We define the women of interest in this study—those aged 14–19 and 20–24 years old—as ‘young mothers’ as per the World Health Organization’s (WHO’s) definition^14^.

## Methods

### Cohort selection

We used the Cebu Longitudinal Health and Nutrition Survey (CLHNS) conducted in Cebu City, Philippines^15^. It is a three-generation community-based cohort, comprised of households from four urban and seven surrounding rural areas. Using the Philippine’s 1980 census as the sampling frame, a single-stage cluster sampling technique was employed to randomly select *barangays* (basic geographical, administrative units in the Philippines). This survey recruited 3,327 pregnant women, which is representative of women of reproductive age in Cebu City. From this, 3,080 women aged 14–47 years old with singleton livebirths were included in the final sample for follow-up. Details about CLHNS sampling technique were discussed in a separate paper^15^.

In this study, we used the 1983–1986 CLHNS data which consist of the baseline and bimonthly follow-up information of women surveyed. While this dataset describes young mothers 35 years ago, results from this study are still relevant in the Philippines and other developing countries due to consistent trends of adolescent fertility and repeated adolescent pregnancy as well as patterns of poor infant feeding habit among young mothers across years^12, 16, 17^. Baseline data, which include pregnancy history, household demographics, and socio-economic status were collected during the second to third trimester of pregnancy, followed by an immediate postpartum interview using a validated questionnaire^15^. Afterwards, bimonthly data collection for 24 months was conducted to follow the health and nutritional status of the mother and the index child.

In the case of this study, we only used the data collected at 12 and 24 months. Maternal height and infant length were measured using calibrated meter sticks and infantometers^15, 18^. Data collectors were trained and assessed for proficiency using the Habicht procedure^19^. We used data from 1,284 mothers aged 14–24 years and their index children who had complete data at the 12- and 24-month data collection points. The retention rate was 88% at the 24-month follow-up.

### Outcome measure

We used the WHO’s 2007 growth standard to derive the length-for-age *z*-score (LAZ) of the index child^20^. LAZ was calculated by dividing the difference between the observed value and the mean value of the reference population by the standard deviation of the reference population. Calculation was automated in Stata using the *zanthro* macro^21^.

Using this score, we defined stunting as LAZ < − 2 at 12 and 24 months. We created a measure of persistence of stunting from 12 to 24 months based on classifications developed by previous studies^22^. Index children who were stunted at both 12 and 24 months were classified as “persistent”; those stunted at 12 months but not at 24 months were classified as “recovered”; those stunted only at 24 months were classified as “late incident”; and those who did not experience stunting were classified as “normal”.

### Exposure

We used the number of past pregnancies in reference to the index child to measure repeated pregnancies. This means that children from a repeated pregnancy are born to young mothers who have at least one past pregnancy. In our analyses, we considered repeated pregnancy as a count variable to enable comparison in an ordinal approach.

We adjusted our analyses for child’s sex, maternal height, occurrence of pregnancy complications, frequency of antenatal visits (i.e. did or did not have ≥ 4 antenatal visits starting 1st trimester), and occurrence of infant diarrhea within seven days before the survey. We also measured and adjusted our analyses for socio-economic factors: maternal and paternal education and employment (i.e. employed or unemployed), and income class, at baseline and during 12-month follow-up. Instead of using all levels of education, we categorised maternal and partner’s education into completion and non-completion of secondary education. We used the monthly household income from all sources and created three income classes using Cebu’s average household income^23, 24^.

### Statistical analyses

Univariate linear and logistic regression analyses were used to assess associations between repeated pregnancy and stunting outcomes: LAZ and stunting occurrence at 12 and 24 months, and stunting persistence. Multivariable models were used to adjust for confounders mentioned above. To measure the relationship between repeated pregnancy and stunting persistence, we used multinomial logistic regression since persistence has four possible discrete outcomes (i.e. persistent, recovered, late incident, and normal). Regression coefficients in this analysis were expressed as relative risk ratio. We compared the risk of stunting persistence among children of women who had more repeated pregnancies with children of women who had no or fewer repeated pregnancies.

Mediation tests were conducted via low birthweight and poor feeding practices since low birthweight (i.e. < 2,500 g) is on the causal pathway between parity and stunting, and feeding practice variables are likely to reduce the risk of child stunting (S1). We used four binary (yes or no) predictors to represent feeding practices at birth, birth to 6 months, 6 to 8 months, 12 months: initiation of breastfeeding within 24 h after delivery, consistent breastfeeding for 6 months after birth, complementary between 6 and 8 months, and breastfeeding at 12 months. Our operational definition is adapted from the indicators set by the WHO to assess infant feeding practices^25^.

Among feeding practice predictors, only breastfeeding at 12 months and complementary feeding were simultaneously analysed as mediators in the models. Initiation of breastfeeding and consistent breastfeeding have null effects to stunting and weak associations with repeated pregnancy which disqualify these variables for mediation testing^26^. We used the *binary_mediation* macro to perform mediation analysis with regression coefficients bootstrapped in 10,000 simulations to obtain robust confidence intervals at 0.05 level of error. This macro adapted the standard Baron and Kenny set of equations to handle binary mediators in our study for continuous and discrete outcomes^27^. Mediated effects of birthweight and feeding practices will be estimated as a proportion by dividing the total indirect effects by the total effect^28^.

All analyses were performed using Stata 14.

### Ethical considerations

Informed consent was obtained from study participants. This study was conducted in accordance with the Australian National Health and Medical Research Council guidelines, the guiding principles for ethical research of the US National Institutes of Health, and the principles of the Declaration of Helsinki.

This study was approved by The University of Queensland School of Public Health Ethics Committee on April 11, 2016. The conduct of the CLHNS surveys were reviewed and approved by the Institutional Review Board of the University of North Carolina at Chapel Hill.

## Results

### Sample characteristics

A total of 1,033 mother–offspring dyads had complete LAZ data at both 12- and 24-month follow-ups. This consists of 299 14–19 year old and 734 20–24-year-old eligible women, most were unemployed (n = 629, 60.8%), did not completed high school (n = 826, 80.0%), and were from middle income class (n = 646, 62.6%) (refer to Table 1). Approximately 40% of the 14–19 year olds and 70% of 20–24-year olds had ≥ 1 pregnancy prior to the index child. More than half of young mothers had consistently breastfed until 6 months (n = 576, 55.8%) while almost all had provided complimentary feeding between 6 and 8 months to the index child (n = 996, 96.5%).

**Table 1:** Sample characteristics.

Measures	Overall (14–24 years old; N = 1,033)	Age group	
		15–19 years old (n = 299)	20–24 years old (n = 734)
**Repeated pregnancies**^**b**^			
0	530 (38.2)	254 (61.5)	276 (28.3)
1	418 (30.2)	120 (29.8)	298 (30.5)
2	256 (18.4)	33 (8.0)	223 (22.9)
3 +	185 (13.3)	6 (1.5)	179 (18.3)
LAZ at 12 months^a^	− 1.69 (1.2)	− 1.79 (1.1)	− 1.65 (1.2)
Stunting at 12 months^b^	405 (37.4)	123 (39.5)	282 (36.8)
LAZ at 24 months^a^	− 2.35 (1.1)	− 2.43 (1.1)	− 2.32 (1.1)
Stunting at 24 months^b^	654 (60.3)	202 (63.3)	452 (59.1)
**Persistence of stunting**^**b**^			
Persistent	348 (33.7)	107 (35.8)	241 (32.8)
Late Incident	275 (26.6)	82 (27.4)	193 (26.3)
Recovered	39 (3.8)	10 (3.3)	29 (4.0)
Normal	371 (35.9)	100 (33.4)	271 (36.9)
**Birthweight**^**b**^			
< 2,500 g	119 (13.5)	58 (22.4)	61 (9.8)
**Index child breastfed within 24 h after delivery**^**b**^			
Yes	412 (39.9)	134 (44.8)	278 (37.9)
**Index child consistently breastfed until 6 months**^**b**^			
Yes	576 (55.8)	160 (53.5)	416 (56.8)
**Index child given semi-/solid foods between 6 and 8 months of age**^**b**^			
Yes	996 (96.5)	291 (97.3)	705 (96.2)
**Index child breastfed at 12 months**^**b**^			
Yes	626 (60.7)	174 (58.2)	452 (61.7)
**Sex of the index child**^**b**^			
Male	534 (51.7)	389 (53.1)	145 (48.5)
Female	498 (48.3)	344 (46.9)	154 (51.5)
**Education attainment of the young mother**^**b**^			
Completed High School	207 (20.1)	46 (15.4)	161 (22.0)
**Income class of the young mother at baseline**^**b**^			
Low	246 (23.8)	92 (30.8)	154 (20.0)
Middle	646 (62.6)	168 (56.2)	478 (65.2)
High	140 (13.6)	39 (13.0)	101 (13.8)
**Employment status of the young mother at baseline**^**b**^			
Employed	404 (39.2)	104 (34.8)	300 (40.9)
Height of the young mother in centimeters^a^	150.31 (5.3)	149.71 (5.0)	150.55 (5.4)
**Number of antenatal visits**^**b**^			
≥ 4 antenatal visits starting 1st trimester	48 (4.65)	6 (2.0)	42 (5.7)
**Complications during delivery**^**b**^			
Yes	134 (13.0)	43 (14.4)	91 (12.4)
**Infant diarrhoea at 12 months**^**b**^			
Yes	242 (23.5)	73 (24.4)	169 (23.1)
**Paternal education**^**b**^			
Completed high school	242 (25.6)	54 (21.1)	18 (27.21)
Paternal age^a^	24.39 (4.2)	22.07 (3.3)	25.25 (4.1)

*LAZ* length-for-age Z-score.

^a^Mean (standard deviation).

^b^Frequency (%).

Compared to offspring of 20–24-year-old women, offspring of 14–19 year olds showed lower average LAZ and higher stunting prevalence both at 12 and 24 months. Children of 14–19 year olds demonstrated an average LAZ of − 1.79 [Standard Deviation (SD) = 1.1] at 12 months and − 2.43 (SD = 1.1) at 24 months; and stunting prevalence of 39.1% and 63.3% at 12 and 24 months respectively. More than a third of the offspring sample showed persistent stunting from 12 through to 24 months of age. These estimates were elevated in reference to the overall values.

### Prevalence of stunting

**Figure 1:**
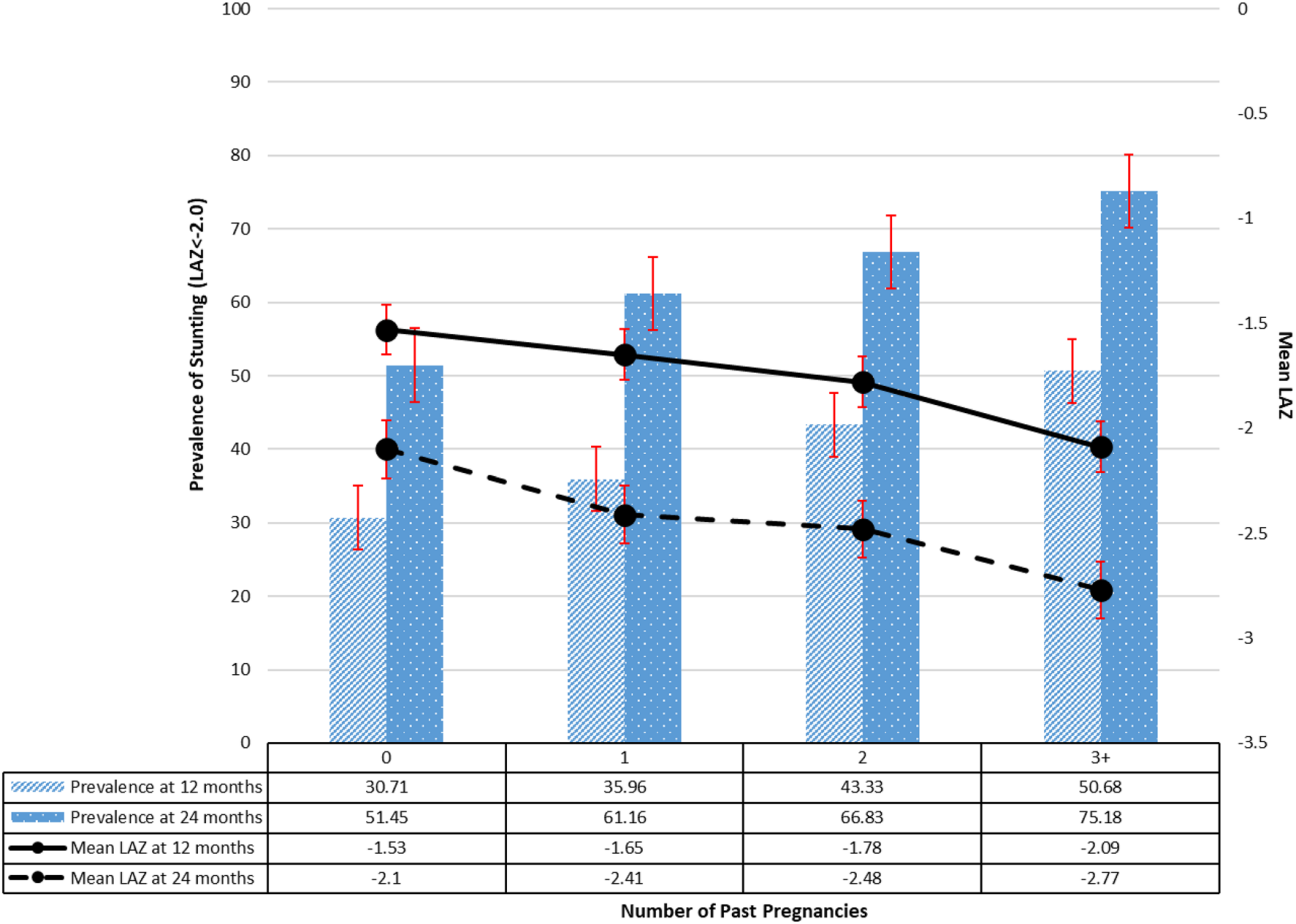
Prevalence of stunting and mean length-for-age z scores (LAZ) at 12- and 24-month follow-up by number of past pregnancies in young mothers.

Offspring of young mothers with repeated pregnancies (i.e. with at least one past pregnancy) showed a higher prevalence of stunting and lower mean LAZ compared with offspring of mothers with no past pregnancies (Fig. 1). There was a large difference in mean LAZ: from − 1.53 (95% CI − 1.64, − 1.41) among offspring of mothers with no past pregnancies to − 2.09 (95% CI − 2.30, − 1.89) among offspring of mothers with ≥ 3 pregnancies at 12 months and; from − 2.10 (95% CI − 2.18, − 1.97) among offspring of mothers with no past pregnancies to − 2.77 (95% CI − 2.98, − 2.59) among offspring of those with ≥ 3 pregnancies at 24 months. We also found that LAZ is slightly lower and stunting prevalence is slightly higher in the 14–19 age group than in the 20–24 age group, particularly at 24 months (S2).

### Occurrence and persistence of stunting among young mothers with repeated pregnancies

Young mothers (14–24 years old) who experienced repeated pregnancies were more likely to have stunted offspring at 12 and 24 months (Table 2). Offspring from a repeated pregnancy showed 40% (OR 1.40, 95% CI 1.19, 1.67) increased odds at 12 months and 25% (OR 1.25, 95% CI 1.04, 1.50) increased odds to be stunted at 24 months. The LAZ at 12 and 24 months in offspring of mothers who had experienced repeated pregnancies was at least 0.15 LAZ units lower compared to those of mothers who had had no previous pregnancies. We also observed a high risk of stunting persistence from 12 to 24 months. Subsequent offspring showed 1.51 times the risk of persistent stunted growth (Relative Risk Ratio 1.51, 95% CI 1.21, 1.88) compared with offspring born to first time mothers.

**Table 2:** Occurrence and persistence of stunting among children of young mothers aged 14–24 years old with repeated pregnancies.

Predictors	Length-for-age Z-score^d^		Stunting occurrence^e^		Persistence of stunting^cf^		
	12 months^a^	24 months^b^	12 months^a^	24 months^b^	Persistent	Late Incident	Recovered
*Number of past pregnancies (repeated pregnancies)*							
Univariate	− **0.15** **(**− **0.21,**− **0.10; < 0.001)**	− **0.20** **(**− **0.26,**− **0.15; < 0.001)**	**1.28** **(1.16, 1.43; < 0.001)**	**1.37** **(1.23, 1.54; < 0.001)**	**1.49** **(1.31, 1.71; < 0.001)**	**1.29** **(1.12, 1.49; < 0.001)**	1.00 (0.73, 1.39; 0.980)
Multivariate	− **0.16** **(**− **0.24,**− **0.09; < 0.001)**	− **0.15** **(**− **0.23,**− **0.08; < 0.001)**	**1.40** **(1.19, 1.67; < 0.001)**	**1.25** **(1.04, 1.50; 0.016)**	**1.51** **(1.21, 1.88; < 0.001)**	1.12 (0.90, 1.40; 0.298)	1.40 (0.89, 2.18; 0.122)
*Low birthweight*							
Univariate	− **0.69** **(**− **0.90,**− **0.48; < 0.001)**	− **0.68** **(**− **0.89,**− **0.46; < 0.001)**	**2.49** **(1.68, 3.68; < 0.001)**	**2.42** **(1.56, 3.77; < 0.001)**	**3.29** **(2.02, 5.37; < 0.001)**	1.52 (0.86, 2.68; 0.146)	1.09 (0.31, 3.79; 0.894)
Multivariate	− **0.57** **(**− **0.79,**− **0.36; < 0.001)**	− **0.49** **(**− **0.71,**− **0.78; < 0.001)**	**2.52** **(1.55, 4.10; < 0.001)**	**2.10** **(1.23, 3.59; 0.006)**	**3.41** **(1.83, 6.37; < 0.001)**	1.35 (0.70, 2.62; 0.370)	1.05 (0.27, 4.04; 0.939)
*Index child breastfed within 24 h after delivery*							
Univariate	− **0.20** **(**− **0.34, 0.07; 0.004)**	− **0.21** **(**− **0.35,**− **0.07; 0.003)**	**1.40** **(1.08, 1.81; 0.011)**	**1.67** **(1.06, 1.77; 0.018)**	**1.55** **(1.15, 2.10; 0.004)**	1.19 (0.86, 1.64; 0.282)	1.15 (0.58, 2.27; 0.680)
Multivariate	− 0.12 (− 0.27, 0.03; 0.121)	− 0.04 (− 0.19, 0.11; 0.583)	**1.42** **(1.01, 2.03; 0.045)**	1.06 (0.75, 1.50; 0.759)	1.35 (0.89, 2.06; 0.163)	0.87 (0.58, 1.32; 0.523)	1.36 (0.57, 3.26; 0.485)
*Index child consistently breastfed until 6 months*							
Univariate	0.10 (− 0.04, 0.23; 0.169)	− 0.03 (− 0.16, 0.11; 0.695)	0.85 (0.65, 1.09; 0.196)	**1.35** **(1.05, 1.73; 0.019)**	1.09 (0.81, 1.46; 0.561)	**1.61** **(1.17, 2.22; 0.003)**	**0.63** **(0.32, 1.23; < 0.001)**
Multivariate	− **0.34** **(**− **0.68,** − **0.01; 0.048)**	− **0.51** **(**− **0.84,** − **0.18; 0.003)**	1.69 (0.74, 3.89; 0.214)	**3.02** **(1.41, 6.49; 0.005)**	**3.97** **(1.34, 11.72; 0.013)**	1.86 (0.78, 4.44; 0.165)	0.30 (0.08, 1.16; 0.081)
*Index child breastfed at 12 months*							
Univariate	0.13 (− 0.01, 0.27; 0.061)	0.01 (− 0.13, 0.15; 0.849)	0.82 (0.63, 1.06; 0.122)	1.23 (0.95, 1.58; 0.115)	1.00 (0.74, 1.34; 0.987)	**1.55** **(1.12, 2.16; 0.008)**	0.84 (0.43, 1.63; 0.609)
Multivariate	**0.65** **(0.30, 0.99; < 0.001)**	**0.73** **(0.39, 1.07; < 0.001)**	**0.34** **(0.15, 0.80; 0.014)**	**0.33** **(.15, 0.72; 0.006)**	**0.17** **(0.06, 0.51; 0.002)**	0.67 (0.27, 1.66; 0.939)	1.63 (0.42, 6.36; 0.482)
*Index child given semi-/solid foods between 6 and 8 months of age*^b^							
Univariate	**0.45** **(0.08, 0.82; 0.017)**	**0.42** **(0.05, 0.78; 0.028)**	**0.41** **(0.21, 0.82; 0.011)**	0.58 (0.27, 1.21; 0.143)	**0.38** **(0.17, 0.89; 0.025)**	0.85 (0.30, 2.36; 0.750)	0.41 (0.08, 2.00; 0.269)
Multivariate	**0.59** **(0.17, 1.00; 0.006)**	**0.68** **(0.27, 1.08; 0.001)**	**0.31** **(0.12, 0.80; 0.015)**	**0.26** **(0.08, 0.82; 0.021)**	**0.19** **(0.05, 0.66; 0.009)**	0.68 (0.17, 2.71; 0.586)	*Not enough cases*

^a^The multivariate model was adjusted for maternal age, maternal height, partner’s age, socio-economic characteristics, offspring diarrhoea at 12 months, pregnancy complications, and antenatal visits, and sex of the index child.

^b^The multivariate model was adjusted for maternal age, maternal height, partner’s age, socio-economic characteristics, offspring diarrhoea at 24 months, pregnancy complications, antenatal visits, and sex of the index child.

^c^The multivariate model was adjusted for maternal age, maternal height, partner’s age, socio-economic characteristics, offspring diarrhoea at 12 and 24 months, pregnancy complications, antenatal visits, and sex of the index child; Reference group for outcome is ‘Normal’.

^d^Estimates are in mean difference (95% confidence interval; *p* value).

^e^Estimates are in odds ratio (95% confidence interval; *p* value).

^f^Estimates are in relative risk ratio (95% confidence interval; *p* value).

Bold values are statistically significant

We found null interactions by maternal age, which suggests no substantive difference between the risk of offspring stunting in women aged 14–19 and 20–24 years (S3). This was also confirmed by similar effect estimates and prevalence differences across number of past pregnancies for each age group.

Low birthweight, introduction of semi-/solid foods between 6 and 8 months (or complimentary feeding) and breastfeeding at 12 months have consistently demonstrated strong association with LAZ and stunting occurrence at 12 and 24 months (see Table 2). These associations were also observed among those who had persistent offspring stunting at both time points.

### Repeated pregnancy and stunting via low birthweight and feeding practice

After confirming a direct effect of repeated pregnancy among young mothers on offspring stunting, we conducted a series of regression analyses to test for mediation via low birthweight and feeding practice predictors. Mediator feeding practices included breastfeeding at 1 year and complementary feeding due to their consistent associations with stunting outcomes. Mediating effects showed that repeated pregnancy via low birthweight decreased LAZ by 0.16 units (95% CI − 0.24, − 0.08) at 12 months and 0.15 units (95% CI − 0.22, − 0.07) at 24 months (Table 3). Analysis using binary stunting outcomes showed that mediation via optimal feeding practices reduced the effects of repeated pregnancy. This is equivalent to 13.66% and 19.95% mediation via feeding practices for stunting occurrence at 12 and 24 months. We only analysed mediation for ‘persistent’ stunting outcome due to the null effects of repeated pregnancy, low birthweight and feeding practices to ‘late incident’ and ‘recovered’ stunting as shown in Table 2.

**Table 3:** Mediated effects of repeated pregnancy in young mothers on stunting via low birthweight and feeding practices.

Outcomes	Via low birthweight			Via feeding practices (combined effect of breastfeeding at 12 months and introduction of semi-solid and/or solid foods between 6 and 8 months only)		
	Total effect	Total indirect effect	%	Total effect	Total indirect effect	%
LAZ at 12 months^ac^	− 0.16 (− 0.24, − 0.08)	0.02 (− 0.01, 0.05)	10.81	− 0.15 (− 0.27, − 0.02)	0.03 (− 0.06, 0.14)	20.13
LAZ at 24 months^ad^	− 0.15 (− 0.22, − 0.07)	0.02 (− 0.01, 0.05)	10.28	− 0.13 (− 0.27, − 0.01)	0.03 (− 0.08, 0.14)	24.78
Stunting at 12 months^bc^	1.22 (1.08, 1.35)	0.98 (0.95, 1.01)	7.87	1.20 (1.02, 1.38)	0.97 (0.86, 1.06)	13.66
Stunting at 24 months^bd^	1.14 (1.01, 1.30)	0.99 (0.95, 1.01)	10.56	1.13 (1.07, 1.27)	0.98 (0.88, 1.07)	19.95
Persistent stunting^be^	1.30 (1.11, 1.49)	0.97 (0.92, 1.01)	9.74	1.26 (1.03, 1.65)	0.98 (0.76, 1.26)	18.09

*LAZ* length-for-age Z-score, %-Proportion mediated.

^a^Adjusted mean difference and 95% confidence intervals.

^b^Adjusted odd ratios and 95% confidence intervals.

^c^Adjusted for maternal age and height, partner’s age, birthweight, initiation of breastfeeding within 24 h after delivery, consistent breastfeeding for 6 months after birth, socio-economic characteristics, diarrhea at 12 months, pregnancy complications, antenatal visits and sex of the index child.

^d^Adjusted for maternal age and height, partner’s age, birthweight, initiation of breastfeeding within 24 h after delivery, consistent breastfeeding for 6 months after birth, socio-economic characteristics, diarrhea at 24 months, pregnancy complications, antenatal visits and sex of the index child.

^e^Adjusted for maternal age and height, partner’s age, birthweight, initiation of breastfeeding within 24 h after delivery, consistent breastfeeding for 6 months after birth, socio-economic characteristics, diarrhea at 12 and 24 months, pregnancy complications, antenatal visits and sex of the index child.

## Discussion

Our study produced robust estimates to show that repeated pregnancy is a predictor for stunting. Our finding contributes to strengthening the limited evidence on the impact of repeated pregnancy as a predictor of child health, with a particular focus on evidence from low- and middle-income countries^29^. We found that children of young mothers with repeated pregnancy are at increased stunting occurrence before the age of two compared to first-time young mothers.

In addition to stunting occurrence at two separate time points, subsequent children also showed higher risk of persistent stunting from 12 to 24 months. This is of particular concern if one considers that children commonly have their best chance of recovering from stunting within the first 2 years of life^18^. Our findings on persistence of stunting during the first 2 years of life is supported by a cross-sectional analysis of 18 countries conducted by United Nations Children’s Fund which showed an increased prevalence and reduced LAZ at 0–11 and 12–23 months among offspring of 15–19 year old mothers^30^. Another multi-country analysis of five cohort studies in developing countries found similar results in its preliminary analysis; a decline in offspring’s LAZ at 2 years by parity^10^. A multi-level meta-analysis also found repeated pregnancies influence delayed infant growth^31^.

The impact of repeated pregnancy on stunting can be explained by the ‘dual-developmental crisis’ experienced by young mothers during their repeated conceptions^32, 33^. The ongoing nutritional requirement of young mothers due to puberty may deplete foetal nutrition causing low birthweight^34^ which we also found to be strongly associated with stunting in our study. Occurrence of another pregnancy may also disrupt young women’s psychosocial adaptation, which may in turn result in poor health-seeking behaviour on pregnancy nutrition^35^, poor infant feeding practices, and food insecurity within the household^36^. Because repeated pregnancies are often unintended^37^, young women may also be at risk of multiple psychosocial disadvantage including educational disruption, inadequate socio-economic resources, and poor human capital^36^. It has also been suggested that maternal inexperience, absence of autonomy, and poor hygiene may lead to suboptimum feeding, a precursor to stunting in offspring^10^.

As mediators, low birthweight and poor feeding practices further increased the harmful effect of repeated pregnancy on infants’ growth. Prevention and mitigation programs, especially in the first 1,000 days, are essential to revert these health and social burdens. Addressing low birthweight and suboptimal feeding practices, which are empirically identified in this study as mediators, may show promise for interventions and ultimately improve offspring’s growth trajectories. Improving young women’s access to modern contraception may also contribute to reduced stunting among their first and subsequent children^38^.

Our study adds to the existing literature through a rigorous method which allowed us to investigate this problem by accounting for the effects of important confounders and by exploring mediators with practical implications. Our study also has some limitations. Our models could not account for potential mediator-outcome confounders such as maternal nutritional intake from diet and supplements, as well as other psychosocial factors. Adjusting for these confounders would further reduce the residual errors and improve the certainty of regression coefficients. We were also unable to dissect feeding practice in terms of timing, amount, frequency, and diversity of solid food introduced which would allow this mediator to better inform promotion strategies. Further, we were not able to account for residual biological confounders which can be addressed through a comparative cluster analysis between the first and second child from a young mother.

Repeated pregnancy in young mothers is a predictor of child stunting. Children of young mothers with repeated pregnancies showed persistent stunting from 1 to 2 years of age which was substantially worsened by low birthweight and suboptimal feeding practices. Further research is needed to investigate and establish causal pathways and trajectories, which may clarify the unique pathogenesis of child stunting among young mothers.

## Supplementary information

Supplementary Figure S1.

Supplementary Figure S2.

Supplementary Table S3.

## Data Availability

The data can be freely accessed through this link: https://www.cpc.unc.edu/projects/cebu/datasets.

## References

[1] Borghi E, Casanovas C, Onyango A (2017). WHA Global Nutrition Targets 2025: Stunting Policy Brief.

[2] Black RE (2013). Maternal and child undernutrition and overweight in low-income and middle-income countries. The Lancet.

[3] de Onis M, Branca F (2016). Childhood stunting: a global perspective. Matern. Child Nutr..

[4] Georgiadis A, Penny ME (2017). Child undernutrition: opportunities beyond the first 1000 days. Lancet Public Health.

[5] Prendergast AJ, Humphrey JH (2014). The stunting syndrome in developing countries. Paediatr. Int. Child Health.

[6] Keusch GT (2013). Implications of acquired environmental enteric dysfunction for growth and stunting in infants and children living in low- and middle-income countries. Food Nutr. Bull..

[7] Nguyen PH (2017). The nutrition and health risks faced by pregnant adolescents: insights from a cross-sectional study in Bangladesh. PLoS ONE.

[8] Das J (2017). Nutrition in adolescents: physiology, metabolism, and nutritional needs. Ann. N. Y. Acad. Sci..

[9] King JC (2003). The risk of maternal nutritional depletion and poor outcomes increases in early or closely spaced pregnancies. J. Nutr..

[10] Fall CHD (2015). Association between maternal age at childbirth and child and adult outcomes in the offspring: a prospective study in five low-income and middle-income countries (COHORTS collaboration). Lancet Glob. Health.

[11] Qu P (2017). Association between the Infant and Child Feeding Index (ICFI) and nutritional status of 6- to 35-month-old children in rural western China. PLoS ONE.

[12] Philippine Statistics Authority & and ICF International (2013). Philippines National Demographic and Health Survey 2013.

[13] Capanzana MV, Aguila DV, Javier CA, Mendoza TS, Santos-Abalos VM (2015). Adolescent pregnancy and the first 1000 days (the Philippine Situation). Asia Pac J. Clin. Nutr..

[14] World Health Organisation (2014). Adolescence: A Period Needing Special Attention.

[15] Adair LS (2011). Cohort profile: the Cebu longitudinal health and nutrition survey. Int. J. Epidemiol..

[16] Maravilla JC, Betts KS, Alati R (2018). Trends in repeated pregnancy among adolescents in the Philippines from 1993 to 2013. Reprod. Health.

[17] Ogbo FA, Ogeleka P, Awosemo AO (2018). Trends and determinants of complementary feeding practices in Tanzania, 2004–2016. Trop. Med. Health.

[18] Adair LS, Guilkey DK (1997). Age-specific determinants of stunting in Filipino children. J. Nutr..

[19] Habicht J-P, Butz WP, Pradilla A, Fajardo LF, Acciarri G, Klein RE (1979). Evaluating the Impact of Nutrition and Health Programs.

[20] World Health Organisation (2006). WHO Child Growth Standards: Length/Height-for-Age, Weight-for-Age, Weight-for-Length, Weight-for-Height and Body Mass Index-for-Age: Methods and Development.

[21] Vidmar SI, Cole TJ, Pan H (2013). Standardizing anthropometric measures in children and adolescents with functions for egen: Update. Stata J..

[22] Mendez MA, Adair LS (1999). Severity and timing of stunting in the first two years of life affect performance on cognitive tests in late childhood. J. Nutr..

[23] Solon O, Floro M (1993). The Philippines in the 1980s: A Review of National and Urban Level Economic Reforms.

[24] Albert JRG, Gaspar RE, Raymundo MJM (2015). Why We Should Pay Attention to the Middle Class?.

[25] World Health Organisation. *Indicators for Assessing Infant and Young Child Feeding Practices: Conclusions of a Consensus Meeting held 6–8 November 2007 in Washington D.C., USA* (World Health Organisaiton, Geneva, 2007).

[26] MacKinnon DP, Fairchild AJ, Fritz MS (2007). Mediation analysis. Annu. Rev. Psychol..

[27] Wiles N, T. L., Abel A, et al. in *Health Technology Assessment* Vol. 18.31 Ch. Chapter 9, Mediated effect of cognitive behavioural therapy on depression outcomes (NIHR Journals Library, 2014). 10.3310/hta18310 PMC4781198 24824481

[28] How can i perform mediation with binary variables? https://stats.idre.ucla.edu/stata/faq/how-can-i-perform-mediation-with-binary-variables/. Accessed 15 Mar 2018.

[29] Fenske N, Burns J, Hothorn T, Rehfuess EA (2013). Understanding child stunting in India: a comprehensive analysis of socio-economic, nutritional and environmental determinants using additive quantile regression. PLoS ONE.

[30] Yu SH, Mason J, Crum J, Cappa C, Hotchkiss DR (2016). Differential effects of young maternal age on child growth. Glob. Health Act..

[31] Danaei G (2016). Risk factors for childhood stunting in 137 developing countries: a comparative risk assessment analysis at global, regional, and country levels. PLoS Med..

[32] Sadler LS, Catrone C (1983). The adolescent parent: a dual developmental crisis. J. Adolesc. Health Care.

[33] Wu G, Bazer FW, Cudd TA, Meininger CJ, Spencer TE (2004). Maternal nutrition and fetal development. J. Nutr..

[34] Borja JB, Adair LS (2003). Assessing the net effect of young maternal age on birthweight. Am. J. Hum. Biol..

[35] Stephenson T, Symonds ME (2002). Maternal nutrition as a determinant of birth weight. Arch. Dis. Child. Fetal Neonatal Ed..

[36] United Nations Children’s Fund (2013). Improving Child Nutrition: The Achievable Imperative for Global Progress.

[37] Aslam RHW (2017). Intervention now to eliminate repeat unintended pregnancy in teenagers (INTERUPT): a systematic review of intervention effectiveness and cost-effectiveness, and qualitative and realist synthesis of implementation factors and user engagement. BMC Med..

[38] Finlay JE (2012). The association of contraceptive use, nonuse, and failure with child health. Int. J. Child Health Nutr..

