# Supplementary material for: Stunting of children under two from repeated pregnancy among young mothers
Source: Sci Rep. 2020 Aug 31;10:14265. doi: 10.1038/s41598-020-71106-7 (PMC7459341; doi:10.1038/s41598-020-71106-7)
Supplement: Supplementary file 1 — Supplementary Figure S1. [file 41598_2020_71106_MOESM1_ESM.pdf]

Stunting of children under two from repeated pregnancy among young mothers

Running title: Repeated pregnancy and child stunting

Joemer Calderon Maravilla[1,2,5] PhD, FRSPH, RN, Kim S. Betts[1,2,3], PhD, MPH, Linda Adair[4], PhD, Rosa Alati[1,2,3], PhD, MApplSc(Health Sc)

1. Institute for Social Science Research, The University of Queensland, Queensland, Australia
2. Life Course Centre, Australian Research Council Centre of Excellence for Children and Families over the Life Course
3. School of Public Health, Curtin University, Australia
4. Carolina Population Center, University of North Carolina at Chapel Hill, Chapel Hill NC, USA
5. Institute of Nursing, Far Eastern University, Manila, Philippines

Corresponding Author:

Joemer Maravilla, PhD, RN, FRSPH

j.maravilla@uq.edu.au

Keywords: Adolescent pregnancy; infant stunting; repeated pregnancy; teenage pregnancy; young mothers

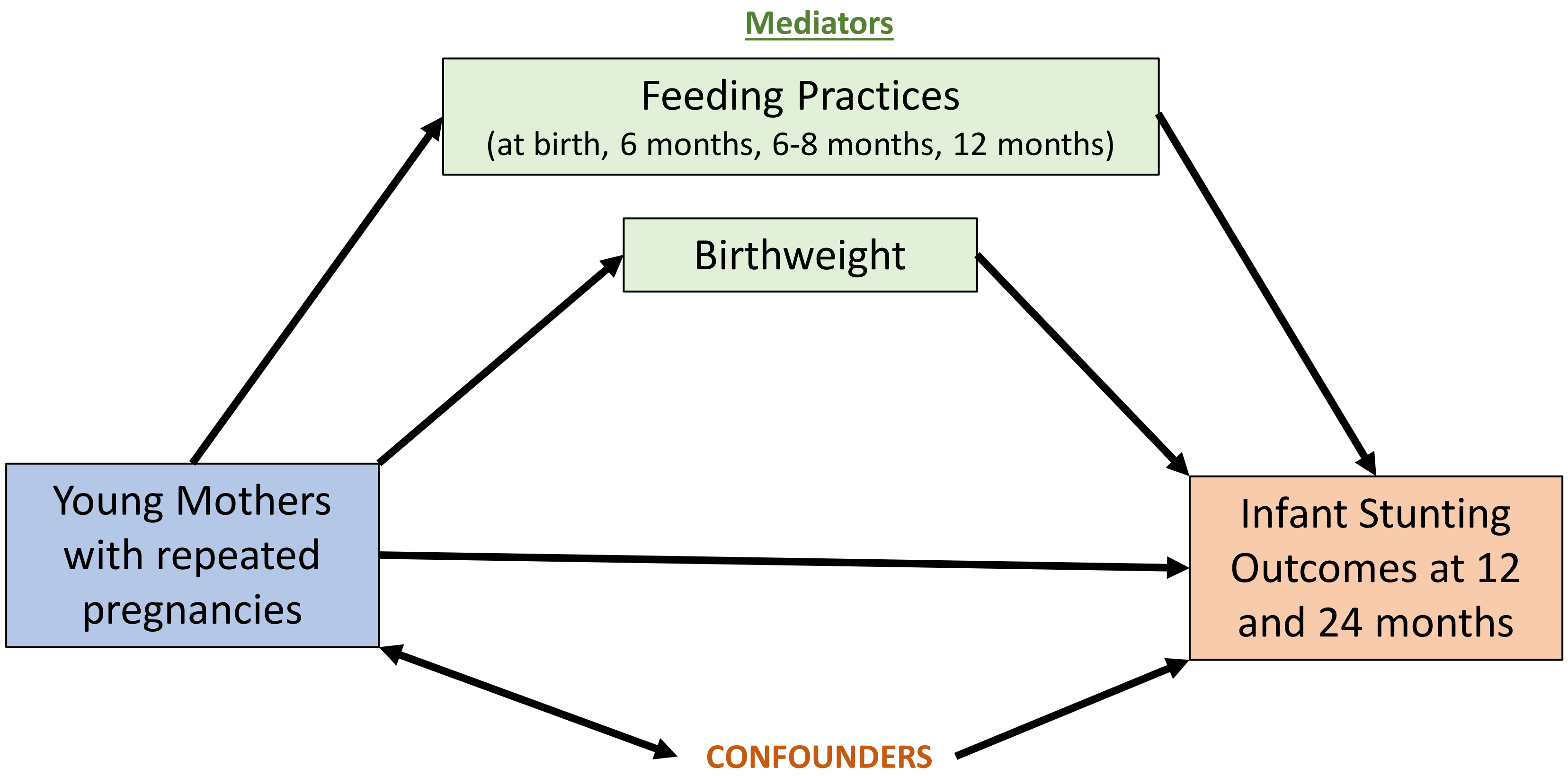

S1. Conceptual Framework
