# Supplementary material for: Stunting of children under two from repeated pregnancy among young mothers
Source: Sci Rep. 2020 Aug 31;10:14265. doi: 10.1038/s41598-020-71106-7 (PMC7459341; doi:10.1038/s41598-020-71106-7)
Supplement: Supplementary file 2 — Supplementary Figure S2. [file 41598_2020_71106_MOESM2_ESM.pdf]

Stunting of children under two from repeated pregnancy among young mothers

Running title: Repeated pregnancy and child stunting

Joemer Calderon Maravilla[1,2,5] PhD, FRSPH, RN, Kim S. Betts[1,2,3], PhD, MPH, Linda Adair[4], PhD, Rosa Alati[1,2,3], PhD, MApplSc(Health Sc)

1. Institute for Social Science Research, The University of Queensland, Queensland, Australia
2. Life Course Centre, Australian Research Council Centre of Excellence for Children and Families over the Life Course
3. School of Public Health, Curtin University, Australia
4. Carolina Population Center, University of North Carolina at Chapel Hill, Chapel Hill NC, USA
5. Institute of Nursing, Far Eastern University, Manila, Philippines

Corresponding Author:

Joemer Maravilla, PhD, RN, FRSPH

j.maravilla@uq.edu.au

Keywords: Adolescent pregnancy; infant stunting; repeated pregnancy; teenage pregnancy; young mothers

S2. Prevalence of stunting and mean length-for-age *z* scores (LAZ) at 12 and 24 month follow-up by number of past pregnancies and age group

(a) 14-19 years old

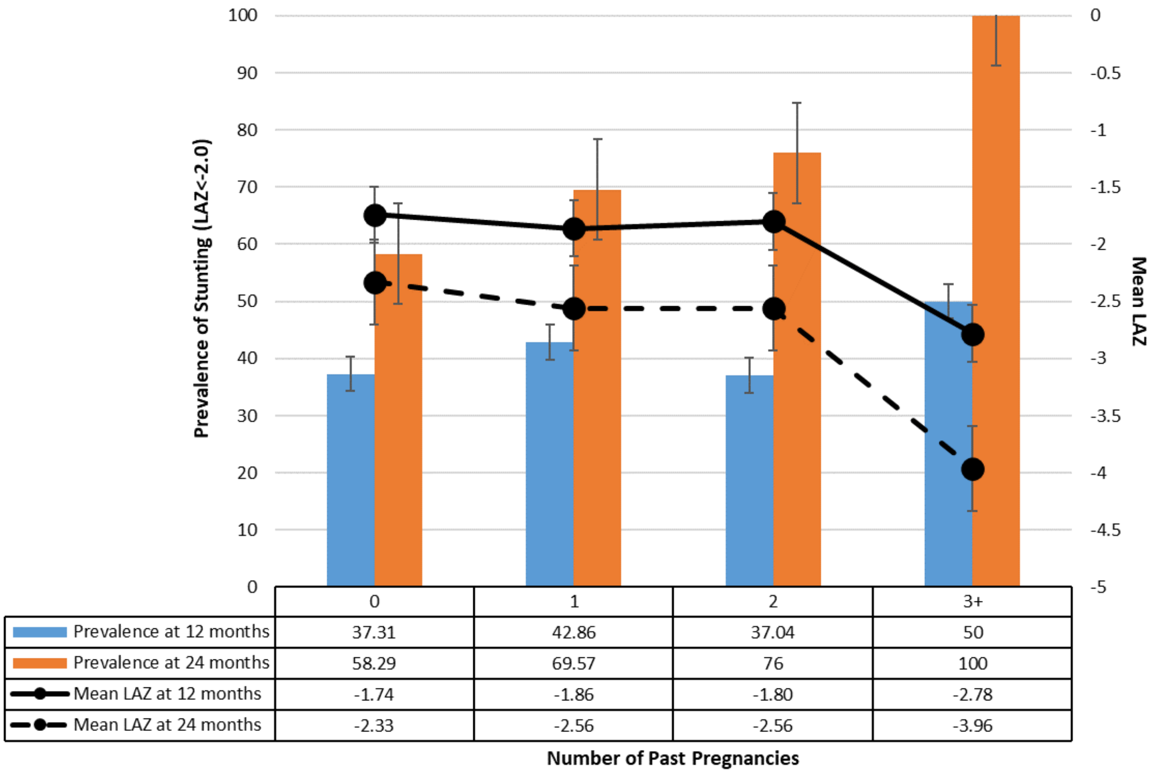

	0	1	2	3+
Prevalence at 12 months	37.31	42.86	37.04	50
Prevalence at 24 months	58.29	69.57	76	100
Mean LAZ at 12 months	-1.74	-1.86	-1.80	-2.78
Mean LAZ at 24 months	-2.33	-2.56	-2.56	-3.96

Number of Past Pregnancies

(a) 20-24 years old

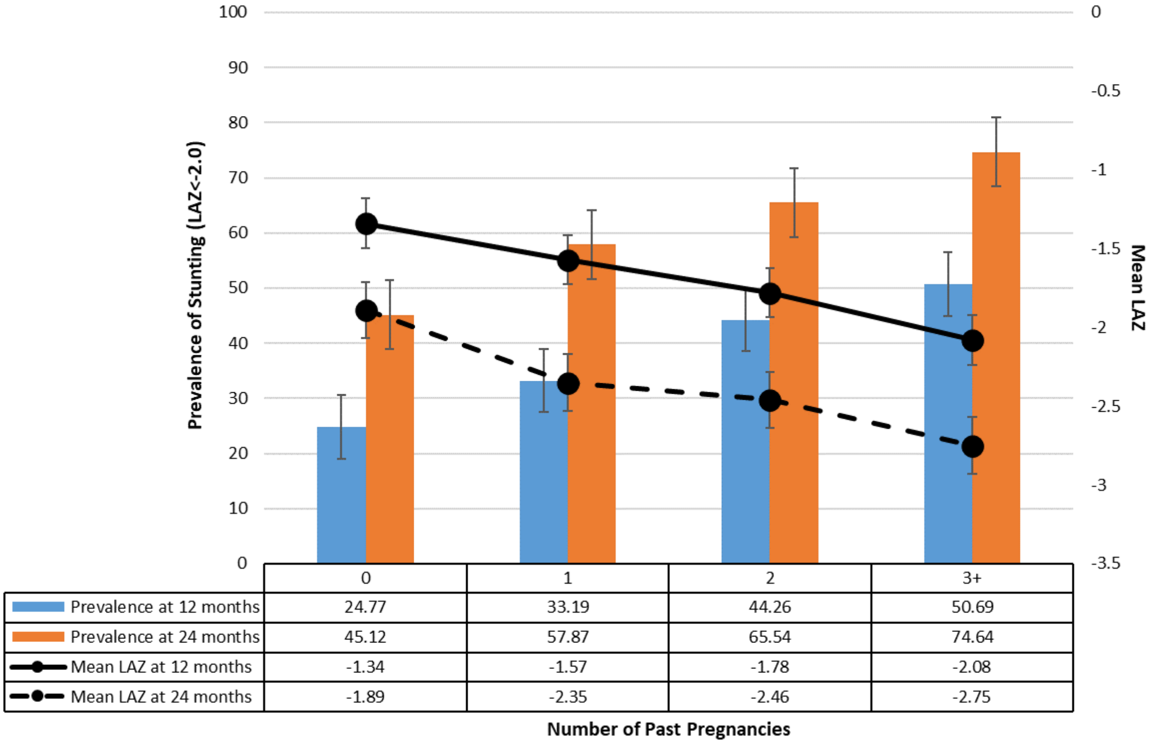

	0	1	2	3+
Prevalence at 12 months	24.77	33.19	44.26	50.69
Prevalence at 24 months	45.12	57.87	65.54	74.64
Mean LAZ at 12 months	-1.34	-1.57	-1.78	-2.08
Mean LAZ at 24 months	-1.89	-2.35	-2.46	-2.75

Number of Past Pregnancies
