# Supplementary material for: Stunting of children under two from repeated pregnancy among young mothers
Source: Sci Rep. 2020 Aug 31;10:14265. doi: 10.1038/s41598-020-71106-7 (PMC7459341; doi:10.1038/s41598-020-71106-7)
Supplement: Supplementary file 3 — Supplementary Table S3. [file 41598_2020_71106_MOESM3_ESM.doc]

**Stunting of children under two from repeated pregnancy among young mothers**

*Running title: Repeated pregnancy and child stunting*

Joemer Calderon Maravilla^1,2,5^ PhD, FRSPH, RN, Kim S. Betts^1,2,3^, PhD, MPH, Linda Adair^4^, PhD, Rosa Alati^1,2,3^, PhD, MApplSc(Health Sc)

1. Institute for Social Science Research, The University of Queensland, Queensland, Australia
2. Life Course Centre, Australian Research Council Centre of Excellence for Children and Families over the Life Course
3. School of Public Health, Curtin University, Australia
4. Carolina Population Center, University of North Carolina at Chapel Hill, Chapel Hill NC, USA
5. Institute of Nursing, Far Eastern University, Manila, Philippines

**Corresponding Author:**

Joemer Maravilla, PhD, RN, FRSPH

j.maravilla@uq.edu.au

**Keywords:** Adolescent pregnancy; infant stunting; repeated pregnancy; teenage pregnancy; young mothers

**S3. Occurrence and persistence of stunting in the first 24 months of life of children** of teenage and young adult mothers with repeated pregnancy

| **Outcomes** | **Age Groups** | | | | **Multivariate with RPxAge%** |
| --- | --- | --- | --- | --- | --- |
|  | **14-19 years old** | | | **20–24 years old** |  |
|  | **Univariate** | **Multivariate** | **Univariate** | **Multivariate** |  |
| LAZ at 12 months*^$^ | -0.13  (-0.31, 0.05) | -0.32  (-0.58, 0.06) | **-0.21**  **(-0.27, -0.14)** | **-0.15**  **(-0.23, - 0.07)** | 0.10 (0.347) |
| Stunting at 12 months*@ | 1.12  (0.82, 1.53) | 1.47  (0.81, 2.69) | **1.40**  **(1.17, 1.68)** | **1.41**  **(1.18, 1.69)** | 0.06 (0.764) |
| LAZ at 24 months^+$^ | **-0.26**  **(-0.44, -0.09)** | **-0.37**  **(-0.62, -0.13)** | **-0.25**  **(-0.31, -0.19)** | **-0.13**  **(-0.21, - 0.05)** | 0.16 (0.101) |
| Stunting at 24 months^+@^ | **1.62**  **(1.12, 2.35)** | **2.51**  **(1.17, 5.39)** | **1.47**  **(1.29, 1.68)** | **1.22**  **(1.01, 1.48)** | -0.45 (0.081) |
| Persistence of Stunting^? |  |  |  |  |  |
| Persistent | **1.64**  **(1.05, 2.54)** | **3.29**  **(1.23, 8.84)** | **1.65**  **(1.41, 1.92)** | **1.46**  **(1.17, 1.84)** | 0.68 (0.227) |
| Late Incident | 1.25  (0.44, 3.54) | **2.91**  **(1.12, 7.57)** | **1.30**  **(1.11, 1.54)** | 1.08  (0.85, 1.37) | **0.53 (0.037)** |
| Recovered | **1.95**  **(1.24, 3.05)** | **7.38**  **(1.08, 50.34)** | 0.97  (0.67, 1.39) | 1.36  (0.81, 2.64) | 0.57 (0.293) |

Abbreviations: IPI-Inter-pregnancy interval; LAZ-Length-for-age Z-score; RPxIPI- 2-way interaction between number of past pregnancies and IPI; IPI and Age group

*The multivariate model was adjusted for IPI, maternal age, maternal height, partner’s age, birthweight, feeding practice predictors, socio-economic characteristics, diarrhoea at 12 months, pregnancy complications and antenatal visits

+The multivariate model was adjusted for IPI, maternal age, maternal height, partner’s age, birthweight, feeding practice predictors, socio-economic characteristics, diarrhoea at 24 months, pregnancy complications and antenatal visits

^The multivariate model was adjusted for IPI, maternal age, maternal height, partner’s age, birthweight, feeding practice predictors, socio-economic characteristics, diarrhoea at 12 and 24 months, pregnancy complications and antenatal visits; Estimates are in regression coefficient (95% Confidence Interval); Reference group for outcome is ‘Normal’

$Estimates are in Mean difference (95% Confidence Interval)

@Estimates are in Odds Ratio (95% Confidence Interval)

?Estimates are in Relative Risk Ratio (95% Confidence Interval)

%Interaction term (p-value)
